# Economic burden and catastrophic cost among people living with type2 diabetes mellitus attending a tertiary health institution in south-east zone, Nigeria

**DOI:** 10.1186/s13104-015-1489-x

**Published:** 2015-10-01

**Authors:** Ijeoma L. Okoronkwo, Jessie N. Ekpemiro, Edna U. Okwor, Pat U. Okpala, Florence O. Adeyemo

**Affiliations:** Department of Health Administration and Management, University of Nigeria Nsukka, Enugu Campus, Enugu, Enugu State Nigeria; Federal Medical Centre Umuahia, Umuahia, Abia State Nigeria; Department of Nursing, University of Nigeria Nsukka, Enugu Campus, Enugu, Enugu State Nigeria; Department of Nursing, College of Health Sciences, Ladoke Akintola University of Technology, Osogbo, Osun State Nigeria

**Keywords:** Economic burden, Catastrophic cost, Type2 diabetes mellitus (DM), Socioeconomic status

## Abstract

**Background:**

Diabetes mellitus (DM) is a life-long illness that affects the quality of life, requiring close monitoring and control. Type 2 DM is preventable and controllable but increasing cost of care could hinder access to quality care because of inability to pay leading to high morbidity, mortality and productivity losses. The people living with diabetes mellitus (PLWD) in Nigeria have high risk for high economic burden and catastrophic expenditure not only because they make frequent visits to the health facilities, report late with complications but also pay out of pocket at the point of accessing care. The aim of this study was to assess the magnitude of economic burden borne and catastrophic costs incurred by PLWD in Nigeria.

**Methods:**

Cross-sectional descriptive survey design was used to study a sample of 308 type2 PLWD managed at a tertiary health institution, South east Nigeria using semi-structured, prevalidated questionnaire. Data collection period was 2 months.

**Results:**

The major findings were economic burden of type 2 DM of N56,245 ($356). Catastrophic direct cost was 45 % at 30 % threshold (the determinant level for catastrophic spending set). All socio-economic status (SES) groups suffered catastrophic expenditure but the poorest quartile had the highest incidence.

**Conclusions:**

Economic burden of DM was high for PLWD who also suffered high catastrophic costs due to the impact of out of pocket payment. PLWD need financial protection especially for the poorest since they buy from the same market and incur same costs. Policy decision making to assist the PLWD cope with cost of care is needful in Nigeria and nations with related problems.

## Background

Diabetes mellitus (DM) is a group of chronic medical conditions in which the body’s metabolism is deranged either due to absence or insufficient production of insulin or the body does not properly respond to insulin; producing a persistent hyperglycaemic state [1]. The persistent hyperglycaemia and associated complications demand intensive care and frequent visits to health facilities thus increasing the cost of care.

DM affects quality of life, requires close monitoring and control. Diabetes mellitus is a growing “epidemic and pandemic” [1]. Globally, about 285 million people had DM in 2010, projected to double by 2030 [2]. Nigeria’s diabetes prevalence of 20.8 million (7 % of population) [3, 4] and her ranking largest in African regional prevalence in 2011 is a great concern [5].

Diabetes is the third cause of death from disease and complications [6], second killer non communicable disease [7], has five fold risks of cardiovascular diseases and three fold of stroke [6]. PLWD in Nigeria have risk for high economic burden and catastrophic expenditure because the three conditions that predict financial catastrophe; healthcare cost paid out of pocket, individuals’ inability to pay and absence of prepayment mechanisms to pool financial risks [8] are prevalent in Nigeria.

Economic burden in this study means direct cost of care. Catastrophic Healthcare expenditure is very high healthcare spending in relation to one’s income beyond which an individual begins to sacrifice consumption of basic needs and use payment coping mechanisms (interium measures to meet up with payment but in the long run increase the total cost). WHO proposed 40 % and above of non-subsistence income consumption (non-food consumption expenditure); that is income available after basic needs have been met but countries could set their thresholds based on their peculiarities [7]. Non-food consumption expenditure is used as proxy to income because it is sensitive; availability of cash and income information are not easily and accurately declared in African countries [9, 10].

Previous studies put catastrophic level at 5–40 % [7–12]. In Nigeria private funding is more than 90 % [9]. More than 70 % of the population live below $1 a day [12, 13] and prepayment mechanism for pooling risk is lacking [9]. Economic impact of healthcare expenditure on individuals challenged with illness especially where prepayment system is absent is a growing concern [14]. Every year more than 150 million individuals in the world face financial catastrophe and more than 100 million individuals are pushed into poverty as a direct result of paying for health care [7, 8, 14]. This could be worse for patients living with Diabetes Mellitus in Nigeria, who receive frequent treatment paid out of pocket coupled with increasing healthcare costs. The burden borne also depends on the amount and level of care received [15], purchasing power of individuals and social insurance policies of that nation [16]. One is bothered that despite the fact that UN in 2011 raised the status of non-communicable diseases (NCDs) to that of HIV/AIDS, TB and Malaria because of their economic and health importance [7, 17], there is neither support, nor financial risk protection (exemption) for DM which is presently assuming an epidemic proportion [2, 5] and presence of development partners and non-governmental organisation (NGOs) have not been felt in DM care [17]. The economic importance, complications and death tolls are compelling national governments to pay more attention to the impacts of DM [7, 17, 18] especially with its late diagnosis in Nigeria and some other Sub Saharan African countries [19–21].

Evidenced-based data is needed to move DM into the health policy agenda of these countries for targeted intervention. Unfortunately, there is paucity of data on the magnitude of the economic burden borne and catastrophic costs incurred by the diabetic Patients in Nigeria. The specific objectives of this study assessed;

1. The direct cost borne by PLWD type 2 in treating diabetes.
2. The catastrophic costs incurred by different SES groups of PLWD attending a tertiary health institution in South-East Nigeria with a view of reducing the economic burden through appropriate decision making.

## Methods

### Study design and setting

A cross sectional descriptive survey design was used for this study. This was considered appropriate because the ‘cost-of-illness estimate’ is a descriptive economic method often used to estimate cost of a particular disease [22].

The study was done in a Sate, South East Nigeria, covering an area of 5834 sq km, 5.8 % of total land mass of Nigeria and has a population of 2,833,999 [23]. It is both a residential and commercial city and has both urban and rural dwellers. The area is made up of civil servants, traders and farmers. The hospital serves both self and health system referred patients. This study was approved by the Ethics Research Committee of Federal Medical Centre (FMC) Umuahia (FMC/QEH/G.596/VOL.4/006).

### Study participants

Population for this study were all type2 DM patients receiving treatment for diabetes mellitus in the institution. An average of 1363 type 2 PLWD received care from the centre in the last 1 year, (diagnostic index 2009–2011 of the Health Records Department) 139 were admitted and managed as in patients while 1224 PLWD were attended to as out-patients (target population). Eligibility criteria were age between 31 and 65 years, receiving treatment in the centre for the past 1 year (July 2011–June 2012) and within the 2 months data collection period, should be actively involved in the management of the condition, well informed about the cost of care (have adequate knowledge of cost of care by self report) and willing to participate in the study.

### Study size

The minimum sample size of 308 was calculated using the formula for estimating a single finite proportion n = Z^2^ × p (1 − p)/d^2^ (Isangedihi et al. 2004) from a target population of 1224 physician diagnosed type2 diabetes mellitus. Due to lack of information on the proportion of type2 DM in economic burden studies, 50 % or 0.5 was chosen as p giving an initial sample size of 384.

2nd stage: In order to obtain the finite population correction for proportion for a small population the formula n = n_0_/1 + (n_0_ − 1)/N was used to get 293.13.

3rd stage: Since data were to be collected directly from the respondents, it was anticipated that as many as 5 % might withdraw from the study prior to its completion through possible refusal to continue with the study. Thus the formula q = n/1 − f where q is the adjustment factor and f is the estimated non response rate was used [ANGEL (n.d.).STAT 509] was used. Therefore the final sample size was 308. However 292 copies of the questionnaire were found to be correctly and completely filled while 16 proposed participants declined.

The Clinics’ appointment registers were used to form a sampling frame. The sampling interval for sample recruitment was 3.97. Therefore every 4th person was systematically recruited on each clinic day till 308 respondents that met the eligibility criteria were selected.

### Variables

The variables of interest were: economic burden, catastrophic diabetic cost, and socioeconomic status. Economic burden was measured by the unit cost of all type 2 diabetes out patient services received. Catastrophic diabetic cost was measured by non food consumption expenditure of the respondents (income) plus the direct cost of diabetes. Socioeconomic status groups was measured by number of household items owned by respondents.

### Data collection

A pretested researcher administered semi-structured questionnaire was used. It comprised of three sections with sixteen questions to elicit responses from respondents based on study objectives. Section A was on demographic characteristics of respondents; section B, on economic burden and section C, on socio economic status and catastrophic cost while the coefficient alpha (Cronbach’s alpha) method was used to calculate the internal consistency reliability of the instrument using 30 PLWD from a peripheral clinic. The co-efficient of reliability obtained by sections were 0.40, 0.80 and 0.75(sections A–C), respectively. The low reliability co-efficient of 0.4 from section A (demographic characteristics of respondents) was related to the heterogeneity created by the string (gender). The study focus was not on the influence of demographic characteristics on economic burden nor catastrophic expenditure, therefore it was considered inconsequential to the study outcome. However, the instrument was late updated with outcome of the pre-tested questionnaire. Informed consents of the patients were obtained and assurance of confidentiality of all information received given. Participants were informed that participation is voluntary. The researchers and the three research assistants trained on the purpose of the study and how to administer the instrument collected the data until the 308 respondents who met the inclusion criteria were recruited. The instrument was administered within the hours of 8 am and 1 pm while the patients awaited their fasting plasma glucose results and to see their physicians to avoid disruption of daily clinic activities and ensure good attention from patients.

### Data analysis

The data gathered were collated, cleaned, coded, grouped and subjected to descriptive statistics on Statistical Package for Social Sciences (SPSS) version 16.0 and presented in percentages, frequencies, means and standard deviations.

Data on personal profile were analyzed using frequencies, percentages means and standard deviations. The economic burden (direct cost) of DM was articulated using bottom-up approach to aggregate the mean cost of units of services received through whole encounter with the health facilities using 1 month recall period. The socio-economic status was determined using principle component analysis (PCA) in STATA Software based on ownership of household assets. The assets were ownership of motorcar, motorcycle, radio, refrigerator, television set and bicycle, electric fan and so on. PCA was used to generate an assets based socio-economic index (grouping all the numerous household items into one whole number) which was then used to divide the respondents into four socioeconomic quartiles (q1–q4) of poorest, poorer, poor and least poor. The first component of the PCA was used to derive weights (eigenvectors) for the SES index. The highest weight was assigned to refrigerator 0.601, followed by personal computer 0.57 and so on as in Table 1. Measure of inequality was the ratio of the mean non-food expenditure of the poorest SES group over that of the least poor. Catastrophic DM cost was determined as a proportion of direct DM cost and non-food expenditure at fixed threshold of 30 % for all SES and 40 and 10 % variable threshold for the least poor and the poorest, respectively. The association between catastrophic DM costs and socio-economic status was assessed using proportions.

## Results

Of the 308 questionnaires administered, 292 which were properly completed (a return rate of 95 %) were analysed and the data presented in Tables. The 16 who declined participation reported that they found the asset based questions sensitive. From Table 1, the mean age of respondents was 54 years, with (67 %) within 50–65 years age group. (53 %) of respondents were females. 79 % were married. 41 % had university/college/polytechnic education while 28 (10 %) had no formal education. 73 (25 %) of the respondents were employed by the government, 16 (6 %) were in private sector employment, 57 (20 %) had retired, 92 (32 %) were self-employed, 10 (3 %) unemployed, 33 (11 %) were farmers while 11 (4 %) were housewives.

**Table 1:** Demographic characteristics of respondents n = 292

	Frequency	%
Age group (in years)		
30–40	40	14
41–50	56	19
50–65	196	67
Total	292	100
Mean age	54	
Gender		
Male	136	46
Female	156	54
Total	292	100
Marital status		
Married	231	79
Single	16	6
Divorced	1	0.3
Widow	41	14
Widower	3	1
Total	292	100
Highest educational attainment		
No formal education	28	10
Primary education	76	26
Junior secondary	5	2
Senior secondary	63	22
University/college/polytechnic	119	41
Post graduate	1	.3
Total	292	100
Employment status		
Unemployed	10	3
Govt. employed	73	25
Private employed	16	6
Self employed	92	32
Retired	57	20
Farming	33	11
Housewife	11	4
Total	292	100

Table 2 shows the cost units on which direct cost was calculated. Cost of DM diet ranked highest N28,524 ($181) followed distantly by drug and investigations; N7701 ($49) and N4236 ($27), respectively. The monthly direct cost of DM was N56,245 ($356). In Table 3, the respondents were categorized into four socioeconomic status groups using (PCA) on STATA software. The first component of the PCA was used to assign weights and assets based index developed using household assets owned by the respondents. The highest weight was assigned to refrigerator (.601) followed by personal computer (.57) and so on as in Table 3. The socioeconomic status quartiles were named as poorest (q1), poorer (q2) poor (q3) and the least poor (q4).

**Table 2:** Direct cost of DM per month reflecting unit costs n = 292 N(US$)

Cost units	Mean (${\bar{\text{X}}}$)	Standard deviation (SD)
Folder	20 (0.13)	25 (0.16)
Drugs	7702 (49)	6922 (44)
Lab. tests/investigations	4932 (31)	5628 (36)
Consultation fees	257 (2)	499 (3)
Insurance premium/co-payment	887 (6)	3351 (21)
Transport	999 (6.3)	3073 (19)
Diabetic diet	28,524 (181)	16,070 (102)
Self-monitoring of glucose	3128 (20)	5984 (38)
Insulin syringe/disposables	959 (6.1)	2575 (16)
Extra house helper	1884 (1.2)	4749 (30)
Physiotherapy	253 (2)	2872 (18)
Dressings	399 (3)	1925 (12)
Cost incurred elsewhere same period on DM	3409 (22)	12,797 (81)
Cost of DM related diseases	2894 (18)	5934 (38)
Total	56,245 (356)	28,907 (183)

**Table 3:** Categorization of respondents into socioeconomic status on assets based index n = 292

Household item	Weight	Yes	No
Radio	.53	284 (97 %)	8 (3 %)
Television	.24	277 (95 %)	15 (5 %)
Air conditioner	.48	29 (10 %)	262 (90 %)
Bicycle	.003	19 (6 %)	273 (94 %)
Motorcycle	−.012	25 (9 %)	267 (91 %)
Car	.34	106 (36 %)	186 (64 %)
Refrigerator	.601	169 (58 %)	123 (42 %)
Power generating set	.29	182 (62 %)	110 (38 %)
Gas cooker	.07	75 (26 %)	217 (74 %)
Electric fan	−.033	254 (87 %)	38 (13 %)
Microwave oven	.18	39 (13 %)	252 (87 %)
Washing machine	.30	14 (5 %)	276 (95 %)
Personal computer	.57	32 (11 %)	257 (89 %)

Socioeconomic status	Quartiles	Frequency	Percentage
Least poor	Q4	72	24.7
Poor	Q3	73	25
Poorer poor	Q2	73	25
Poorest	Q1	74	25.3
Total		292	100

Table 4 showed the mean respondents’ expenditure on food and non-food per month. DM costs as a proportion of non-food expenditure (N256,000) ($1620) was 20 %. Catastrophic expenditure was 45 % at 30 % threshold. In Table 5, the mean non-food expenditure was N256,000 ($1620). The poorest (q1) spent N24,617 ($156) and the least poor group expended N120,228 ($761) on non-food. The ratio of q1/q4 was 1:4.9. The non-food expenditure of the least poor was 4.9 times that of the poorest group. At 30 % fixed threshold, the catastrophic levels were 78, 47, 52 and 39 % (q1–q4), respectively while at variable threshold of 10 and 40 % threshold (q1 and q4), the costs were 86 and 17 %, respectively. Majority that suffered catastrophic expenditure at 30 % threshold belong to the poorest SES group 58 (78 %) followed by the poor (q3) 38 (52 %). All SES groups experienced catastrophic expenditure at 10 % threshold but the poorest was worst affected 64 (86 %) followed by the poorer poor 56 (79 %). Direct cost as a proportion of non-food consumption was 20 %.

**Table 4:** Respondents’ monthly expenditure and mean catastrophic DM cost at 30 % threshold N(US$)

Respondents’ expenditure	Mean (${\bar{\text{X}}}$)	Standard deviation (SD)
Food purchased	31,800 (201)	39,629 (251)
Food produced	9924 (63)	15,233 (96)
Clothing	28,300 (179)	33,884 (215)
Rent	24,400 (154)	53,672 (340)
Health care	15,400 (98)	22,679 (144)
Cooking fuel	5272 (33)	6766 (43)
Educational expenses	84,800 (537)	133,723 (846)
Durable goods	64,001 (405)	22,855 (145)
Community welfare	11,800 (75)	22,332 (141)
Transportation	12,000 (76)	14,879 (94)
Utilities	10,026 (64)	12,214 (77)
Non-food expenditure	256,000 (1620)	731,710 (4631)
Total expenditure	553,725 (3505)	999,078 (6323)
DM costs as a proportion of non-food expenditure	20.4 %	11 %
Catastrophic DM cost	45 %	88 %

**Table 5:** Respondents’ expenditure per month by socioeconomic status, catastrophic expenditure and the monthly mean cost of treating DM at the centre in N(US$)

Expenditure	Q1 the poorest n = 74	Q2 the poorer poor n = 73	Q3 the poor n = 73	Q4 the least poor n = 72
Non-food expenditure	24,616 (156)	43,166 (273)	67,991 (430)	120,228 (761)
Ratio of non-food (qn/q1)	1	1.75	2.76	4.88
Ratio qn/q4	0.20	0.36	0.57	1
Catastrophic threshold				
Threshold of 40 catastrophic	42(57 %)	20(27 %)	13(18 %)	12(17 %)
Threshold of 30 catastrophic	58(78 %)	34(47 %)	38(52 %)	28(39 %)
Threshold of 10 catastrophic	64(87 %)	56(78 %)	50(69 %)	25(35 %)
Mean cost of treatment of DM at the centre	56,245 (356)	56,245 (356)	56,245 (356)	56,245 (356)

## Discussions

The economic burden and catastrophic expenditure among type2 PLWD in this study was found to be high. Worse still, for the lowest SES (q1). All SES groups had high catastrophic cost but the poorest are worse off. The mean monthly direct cost of N56,245 (US$ 356) per PLWD is considered so high for respondents’ mean non-food consumption expenditure of N256,000 (US$1620).

DM cost is more than twice non-consumption expenditure of q1 N24,617 (US$ 156) and more than 43 % that of q4 N120,228 (US$ 761). DM cost in this study appears higher than in previous studies with (US $ 197) per annum in outpatient study in Karachi Pakistan in 2007 [24] and $ 2144.2 per PLWD per year in a West African region study in 2009; [25] but lower than N180,582 (US$1143) in cost of DM foot ulcer [19] in Nigeria 2007.

Diet ranking is highest followed by drugs and investigations differed from previous studies where drugs ranked highest. This high cost may not be unrelated to increasing healthcare cost, high cost of diabetic supplies, frequent visit to hospitals, wide claims on DM diet for “cure”, on media and streets by traditional healers, late reporting with complications and co-morbidities that are known to increase cost burden of DM [18, 22, 26]. High cost of care is likely to impede effective DM care due to low purchasing power, out of pocket payment at the point of need and absence of prepayment mechanisms [12]. Lack of access to effective DM could expose patients complications that will reduce the quality of life, increase morbidity, mortality and productivity loses which has far reaching implications for development. The only way to explain how they paid this huge direct cost is use of payment coping mechanisms; interim measures to meet up with health cost (social support, borrowing, sale of assets, cutting back on consumption of basic needs, skipping appointments or abandoning treatment when feeling well, instalment payment, etc. [12, 27, 28]. Unfortunately financing health care with payment mechanisms further inflates the total costs and push the patients deeper into ‘hidden’ poverty [28]. Catastrophic direct cost was 45 % at 30 % threshold implying that 45 % of the 292 respondents, spent 30 % and above of their non food consumption expenditure (income) on diabetic care.

The catastrophic DM expenditure at 30 % threshold within the SES quartiles (q1–q4) of 58 (78 %), 34 (47 %), 38 (52 %) and 28 (39 %), respectively is considered quite high especially for the poorest with meagre income but must buy at same rate with rich at the facility or forfeit treatment. Previous studies had described this catastrophic cost as financial expenditure that is crippling to household and disrupts their standards of living [29, 30].

Using fixed threshold could mask degree of financial catastrophe and the inequity in financial access to care between poorest and least poor [11, 15]. For example at 30 % threshold in the present study, catastrophic cost was high among all the socioeconomic status groups. The remaining 70 % of non-food expenditure would amount to N17,232 ($109) for the poorest and almost five times higher for the least poor, N84,160 ($533). Thus the least poor cannot be said to be facing as great catastrophe as the poorer ones. The least poor could cut back on his luxuries to cope but the poorest is left with only N17,232 ($109) to survive on. The use of variable threshold to measure catastrophe gave a higher overall and disaggregated levels of catastrophe which is considered a better measure reflecting the differences in purchasing power between the rich and the poor [11].

For example at a variable threshold of 10 % for the poorest and 40 % for the least poor, the catastrophic costs were 64 (87 %) and 12 (17 %), respectively. At the expenditure of 10 % non food more than 80 % experienced financial distress while the least poor will spend almost five times of the same and only 17 % will experience catastrophe. The richest quartile would need to spend about 40 % of their non-food consumption on DM before having the risk of being tipped into poverty while the poorest quartile would need to spend 1/10 that of the richest to be thrown into poverty. Measure of inequality is the ratio of q1/q4 non-food expenditure 1:4.9. Catastrophic cost in this study were higher than previous studies. Although there was no Nigerian Catastrophic DM cost study found in literature other cost of illness studies (TB, Malaria, etc.) in Nigeria demonstrated high catastrophic cost. Onoka and colleagues examined catastrophic health expenditure in Nigeria at variable and fixed thresholds; observed 14.8 % catastrophe at 40 % non-food expenditure with 22.6 and 7.6 % of the poorest and richest, respectively. At a variable threshold of 5 and 29.6 % for the poorest and the richest the catastrophic level were 44.7 and 12.0 % [12]. However Ichoku and colleagues observed, 3–5 % catastrophic spending at 10 % threshold [10]. In a household survey of catastrophic health expenditure in South east Nigeria (n = 167) highest proportion was noted among the poorest [31]. At a variable threshold of 5 and 29.6 %, (q1 and q4) catastrophic level were 44.7 and 12 %, respectively [31].

As revealed in studies (11, 12, 13) it was not somewhat a surprise to find catastrophic expenditure in a country that has the highest DM burden in Africa with no prepayment mechanism to pool financial risk, >70 % of the population live below $1 per day, >90 % use private funding for health services and late reporting for care. However, it becomes a great concern considering that PLWD will need to contend with these costs throughout lifetime.

Secondly the magnitude of economic burden and catastrophic cost observed in this study in South east Nigeria is major concern because, the area is one of the oil producing states with better resource allocation and the National Health Insurance Scheme which presently covers some categories of government workers, is being implemented.

The disproportionate, experience of catastrophe tilting towards lower socioeconomic groups in the present study means that the poor is not protected from impact of out of pocket spending (OOPS) and increasing care cost (inequity) [32].

Urgent re-visitation of the current health financing strategy to provide for financial risk protection of the poor through policy decision making is needful to reduce economic burden of DM. Government should adopt financial strategies that rely less on private funding.

### Study limitations

The study has some limitations. Due to the 1 month recall some cost items may be missed due to forgetfulness. Another limitation is that cost estimation may risk double counting because of cost of co-morbidity (cost-of-illness problem). Multiple accesses for care within the period of study may lead to loss of unit costs. Furthermore self-report of costs can give an underestimation or exaggeration of the problem. Asset based information was observed to be sensitive to some respondents. The study was confined to only one hospital in the state therefore findings cannot be generalized. There was no Nigerian patient-based cost of DM and catastrophic expenditure out-patient study cited as non was seen in literature review.

## Conclusions

This study demonstrated a high economic burden of type2 diabetes mellitus that led to catastrophic expenditure especially among the lowest socioeconomic status group. Consistent with previous studies, the results illustrate the difference in level of catastrophic spending. This calls for re-visitation of the present health financing policy to accommodate pro-poor policies that will make DM care free at the delivery point, subsidize costs or provide a wider coverage of National Health Insurance.

## Abbreviations

$: US dollar
N: Naira
AIDS: acquired immune deficiency syndrome
DM: diabetes mellitus
HIV: human immunodeficiency virus
OOPS: out of pocket spending
SES: socio-economic status
TB: tuberculosis
UN: United Nations
WHO: World Health Organisation
